# Editorial: Epigenetics of inflammatory reactions and pharmacological modulation

**DOI:** 10.3389/fphar.2024.1505196

**Published:** 2024-10-28

**Authors:** Amedeo Amedei, Cinzia Parolini

**Affiliations:** ^1^ Department of Experimental and Clinical Medicine, University of Florence, Florence, Italy; ^2^ Interdisciplinary Internal Medicine Unit, Careggi University Hospital, Florence, Italy; ^3^ Department of Pharmacological and Biomolecular Sciences, Università degli Studi di Milano, Milano, Italy

**Keywords:** epigenetic, inflammatory response, mouse model, pharmacoligical interventions, signalling

Epigenetics is the scientific investigation of how lifestyle and environment can lead to alterations that impact gene function. Unlike genetic changes, epigenetic changes are reversible and do not change the DNA sequence but alter DNA accessibility and chromatin structure, thereby influencing patterns of gene expression (Handy et al., 2011). Several lines of evidence have documented that epigenetic modifications, such as DNA methylation, histone alterations (mainly acetylation, methylation and phosphorylation), and non-coding RNA expression (Goldberg et al., 2007), play a critical role in the normal development and differentiation of various cell lines in the adult organism. Notably, epigenetic programming significantly impacts the regulation of pluripotency genes, which become inactivated during differentiation, and gene expression during inflammation and immune responses.

Pharmacological interventions aimed at controlling inflammation and immune responses have undergone significant development in recent years, including biological agents that target specific cytokines and immune cells involved in inflammatory reactions and small molecule inhibitors of specific inflammatory signaling pathways.

The different papers published in this Research Topic describe the interplay between epigenetic modifications and inflammatory cascades in the context of sepsis, osteoarthritis and acute pancreatitis.

Sepsis is a systemic inflammatory syndrome that is caused by an imbalanced host response to an infection (Parolini, 2020a). Sepsis-induced cardiomyopathy (SIC) is one of the most common complications of sepsis and a major manifestation of multiple organ failure (Hollenberg and Singer, 2021). The interleukin (IL)-6/signal transducer and activator of transcription (STAT)3 signaling pathway plays a crucial role in sepsis, modulating the inflammatory response and coagulation (Lei et al., 2021). Although therapeutic outcomes have improved over time, antibiotic resistance and adverse reactions are the leading cause of therapy failure (Strich et al., 2020). Therefore, the discovery of new effective drugs is imperative. ACT001, a water-soluble derivate of micheliolide (Viennois et al., 2014), has shown anticancer, anti-inflammatory and anti-platelet activities (Zhang et al., 2012; Zhang et al., 2022; Wang and Li, 2015). Peng et al., demonstrated that ACT001 was effective in improving the survival of mice from septic shock and in protecting cardiovascular function during sepsis. These effects were, at least in part, mediated by the ability of ACT001 to downregulate the IL-6/STAT3 pathway, eventually leading to a decrease of the IL-6 and tumor necrosis factor (TNF) protein levels. Taken together, these data suggest that ACT001 has a potential clinical application in the management of sepsis by preserving the homeostasis of the circulatory system.

Osteoarthritis (OA) is a common bone disease that poses a significant threat to human wellbeing and vitality. The main clinical symptoms of OA include swelling around the affected joints, stiffness, joint pain and limited mobility (Loeser, 2009). Notably, OA has emerged as the primary cause of disability in older individuals (Silverwood et al., 2015). To date, the true pathogenesis of OA remains unclear. However, various factors have been associated with OA progression, such as obesity, aging, trauma, inflammation, osteoporosis and joint deformities (Katz et al., 2021; Parolini, 2023).

Bergamottin (5-geranoxypsoralen) is a biologically active furanocoumarin, isolated from bergamot oranges and grapefruits, possessing anti-inflammatory, antioxidant and anti-aging properties (Wang et al., 2022). Recently, it has been demonstrated that its anti-inflammatory activities have been mediated by its ability to negatively modulate the nuclear factor kappa B (NF-kB), by inducing Silent Information Regulator factor 1 (SIRT1) (An et al., 2021). SIRT1 is a member of the mammalian sirtuin family of proteins that can deacetylate various histone and non-histone substrates (Jia et al., 2022). Shen et al. demonstrated, using both *in vitro* and *in vivo* approaches, that Bergamottin inhibited IL-1 beta-induced inflammation and extracellular matrix degradation via the Sirt1/NF-kB signaling pathway. Specifically, Bergamottin decreased the expression of matrix metalloproteinase 13 (MMP-13), cyclooxygenase 2 (COX-2), inducible nitric oxide synthase (iNOS) and A disintegrin, and metalloproteinase with thrombospondin motif 5 (ADAM-5).

It has been demonstrated that both DNA methylation and histone modifications together with environmental stimuli are implicated in the pathogenesis of acute pancreatitis (AP), although the specific mechanisms remain poorly understood (Natale et al., 2019; Pedersen et al., 2022; Weiss et al., 2021). Pollin et al. investigated the impact of euchromatic histone-lysine N-methyltransferase 2 (Ehmt2), a transcriptional repressor (Jan et al., 2021), in experimentally induced AP. The results obtained demonstrated that *Ehmt2* inactivation increases the tendency of the normal pancreas to injury-inflammation, suggesting its seminal role in maintaining pancreatic homeostasis and moderating inflammatory responses. In addition, by using conditional *Ehmt2* inactivation in acinar cells, the authors discovered how epigenetic dysregulation within this single cell type can trigger a cascade of events that eventually lead to an amplified injury-inflammation-repair response across the entire organ.

However, inflammation is a conserved process that involves the activation of immune and non-immune cells, and the secretion of nitric oxide (NO) (De et al., 2016) and pro-inflammatory cytokines to protect the host from pathogens and injury, and to support tissue repair and recovery (Parolini, 2023). Nevertheless, unresolved or sterile inflammation is responsible for the promotion of “low-grade systemic chronic inflammation,” a condition characterized by tissue and organ damage, metabolic disorders, i.e., atherosclerosis, type-2 diabetes, and obesity, and an increased predisposition to various inflammation-based diseases, such as neurodegenerative pathologies, rheumatoid arthritis, inflammatory bowel disease and cancer (Varela et al., 2018; Parolini, 2020b; Solier et al., 2023). Different studies have demonstrated that more dietary components can positively impact modifiable risk factors for chronic human syndromes. Additionally, the importance of prebiotics in modifying the gut microbiota (GM) to improve human health is well known (Busnelli et al., 2018). Recently, polysaccharides extracted from edible fungi have been shown to exert immunomodulatory, antibacterial, antioxidant, anti-inflammatory and anti-tumor activities (Maity et al., 2021; Sun et al., 2022). In fact, polysaccharides serve as an energy source for intestinal microorganisms, promoting their proliferation and the production of beneficial compounds. Additionally, the GM flora can metabolize the polysaccharides to generate short-chain fatty acids (SCFAs), mainly acetic, propionic and butyric acids, which possess immunomodulatory and anti-inflammatory effects (De Almeida et al., 2019). Yin et al. have summarized the anti-inflammatory effects and the mechanisms of action of fungal polysaccharides. Ultimately, these data may trigger new investigations to improve the development and use of edible fungi for therapeutic purposes.

In summary, the studies published in this Research Topic have strengthened the crucial and critical relationship between epigenetic modifications and inflammatory-based diseases, contributing to the discovery of new therapeutic tools to improve the clinical outcome of various human pathologies.
